# The Impact of Core Belief Disruption on PTSD Symptoms in Cancer Patients: The Mediating and Moderating Roles of Intrusion and Avoidance Behavior

**DOI:** 10.1007/s11126-025-10179-x

**Published:** 2025-07-01

**Authors:** Fatma Özgün Öztürk, Ganime Can Gür

**Affiliations:** 1 Faculty of Health Science, Department of Psychiatric Nursing, University of Pamukkale, Denizli, 20000 Turkey; 2 Faculty of Health Science, Department of Psychiatric Nursing, University of Pamukkale, Denizli, Turkey

**Keywords:** Avoidance, Cancer, Core beliefs, Hyperarousal, Intrusion, Moderated mediation, Posttraumatic stress symptoms.

## Abstract

Cancer is a life-threatening illness that, beyond its physical consequences, can lead to significant psychological outcomes, including symptoms of post-traumatic stress disorder (PTSD). While core belief disruption has been linked to PTSD, the pathways underlying this relationship remain underexplored. This study aimed to examine the association between disruptions in core beliefs and PTSD symptoms in cancer patients, with a particular focus on the mediating role of intrusion and the moderating role of avoidance behavior. A total of 204 patients receiving oncology treatment participated in the study. Data were collected using the Personal Information Form, the Core Beliefs Scale, and the Impact of Events Scale-Revised. PROCESS macro for SPSS was used for the analysis: Model 4 for simple mediation and Model 5 for moderated mediation. Findings revealed that disruptions in core beliefs were significantly associated with both intrusion and hyperarousal symptoms (*p* < 0.01**)**. Moreover, intrusion was found to fully mediate the relationship between core belief disruptions and hyperarousal. Avoidance behavior moderated this mediating relationship, such that the direct effect of belief disruption on hyperarousal was significant at low and high levels of avoidance, but not at moderate levels. These findings underscore the importance of addressing both cognitive disruptions and avoidance behaviors in trauma-informed interventions for cancer patients. Accordingly, the development of structured, personalized psychoeducational and therapeutic support programs is recommended to enhance psychological resilience and improve the overall quality of life in cancer survivors.

**Supplementary Information:**

The online version contains supplementary material available at 10.1007/s11126-025-10179-x.

## Introduction

Cancer is a significant public health issue globally, characterized by an increasing incidence and mortality rate [1–3]. The prolonged medical processes following diagnosis lead to multifaceted challenges, impacting patients physically, economically, socially, and psychologically. These issues may emerge at the time of diagnosis, during treatment, or even months to years afterward, with many long-term and late-onset problems prominently affecting psychological health [4]. Anxiety disorders, depression, and post-traumatic stress disorder (PTSD) are frequently reported psychological consequences among cancer patients. The prevalence of these conditions varies according to age groups, cancer types, and diagnostic criteria [5–9].

Particularly noteworthy in this context is cancer-related PTSD (CR-PTSD).CR-PTSD prevalence is reported to range widely from 0 to 32% [10–12], primarily due to differences in diagnostic criteria. For instance, DSM-IV considered life-threatening illnesses such as cancer as traumatic events [13], whereas DSM-5 restricted trauma to sudden and catastrophic medical conditions [14]. This revision complicates the classification of cancer experiences as traumatic events, leading clinicians to categorize symptoms primarily under anxiety or adjustment disorders [15]. As emphasized by Kangas (2013), this change in DSM criteria has directly impacted the assessment of trauma symptoms in cancer survivors [16]. Indeed, meta-analyses based on DSM-5 criteria suggest higher PTSD rates among younger patients, those diagnosed with advanced-stage cancer, and those who recently completed treatment [17]. Additionally, pre-existing psychiatric conditions, past traumas, family history of cancer, younger age, and limited social support emerge as significant risk factors [18, 19].

CR-PTSD symptoms include classical PTSD manifestations such as hyperarousal, emotional numbness, avoidance behaviors, and re-experiencing traumatic memories [20–23]. Multiple theoretical frameworks have been proposed to explain CR-PTSD symptomatology. According to the cognitive appraisal-meaning-making model, the personal threat perception triggered by cancer diagnosis, loss of control, and negative future expectations may initiate traumatic responses [24]. The shattered assumptions theory suggests that the disruption of core beliefs, such as “the world is just and predictable,” exacerbates feelings of vulnerability and unpredictability, sustaining traumatic stress [25]. Emotional processing theory emphasizes that inadequately processed traumatic memories and associated avoidance behaviors perpetuate intrusions, thus maintaining perceived threat and chronic stress [26]. Similarly, the fear-avoidance model asserts that heightened threat perceptions reinforce avoidance behaviors, preventing threat extinction. The fear of cancer recurrence model explains chronic vigilance through interpreting normal bodily sensations as potential recurrence signals [27]. Lastly, the psychobiological stress response model proposes that persistent activation of the hypothalamic-pituitary-adrenal axis and inflammatory processes underpin sustained hyperarousal symptoms [28]. This integrative view underscores the necessity of evaluating CR-PTSD’s cognitive, emotional, behavioral, and biological dimensions for comprehensive diagnosis and intervention planning.

This theoretical framework also guides therapeutic approaches for CR-PTSD. Although treatment research remains limited, cognitive-behavioral therapy (CBT) is highlighted among the most effective interventions [19, 29, 30, 31]. The potential for traumatic experiences to disrupt core beliefs is critical in understanding the cancer-PTSD relationship. Emotionally significant events impacting core beliefs may challenge assumptions of world controllability and predictability [32–38]. Epstein (1991) linked PTSD to the inability to validate core beliefs about oneself and the world, and subsequent research has confirmed that trauma can fracture these beliefs [36, 38, 39, 40]. Janoff-Bulman and Frieze notably articulated, “When disaster strikes, our basic assumptions are shattered, protective illusions vanish, and survivors must confront their vulnerability” [41].

Building on this theoretical foundation, our study examines how life-threatening illness experiences disrupt core beliefs, aiming to explore the relationship between core belief disruptions and PTSD symptoms, along with potential mediating variables among cancer patients. Findings from this research may enhance healthcare professionals’ ability to address emotional needs effectively and optimize CBT-based interventions. Consequently, individuals diagnosed with cancer may better manage trauma responses and support their recovery processes.

### Hypothesis 1

Disruption of core beliefs has an indirect effect on the increase of posttraumatic hyperarousal symptoms, and this effect is mediated through posttraumatic experiences of intrusion.

### Hypothesis 2

Avoidance behavior reduces the effect of core beliefs disruption on increasing hyperarousal symptoms. That is, the presence of avoidance behavior reduces the indirect effect of core beliefs on hyperarousal through intrusion experiences.

## Methods

### Study Design

This study was carried out using a descriptive and relational examining model.

### Sample and Setting

Individuals diagnosed with cancer who were hospitalized in Denizli’s Oncology Hospital or attending the outpatient chemotherapy unit were included in the study population.

A sensitivity analysis (linear multiple regression test in G*Power) was performed to examine the capacity to detect the contribution of distribution in core beliefs, intrusion, and avoidance to hyperarousal (power = 0.95, significance level = 0.05, and effect size = 0.15). Based on the 25% withdrawal rate, we generated a sample size of 111. The final sample of 204 cancer patients consented to participate in the study. The study comprised male and female volunteers who were 18 years or older, could answer the questions, had no cognitive or psychiatric issues, and were diagnosed with any cancer.

### Data Collection

Data were obtained using a patient identification form that included the participants’ sociodemographic and clinical characteristics, Core Beliefs Scale (CBS), and Impact of Events Scale-Revised (IES-R). All eligible participants were informed of the study objectives and methodology. Individuals who needed aid with comprehension were helped by the researcher, who read out the questions and explained any unclear areas using alternate words, while maintaining the original meaning. The questionnaires were completed in the study language and took approximately 15 min to complete.

### Data Collection Tools

#### The Personal Informatıon Form (PIF)

The PIF, which the researchers created after conducting a thorough study of the literature, includes details about socio-demographic variables as well as information regarding cancer.

#### The Core Beliefs Scale (CBS)

The purpose of the CBS developed by Cann et al. (2011) is to assess individuals’ fundamental assumptions about the world, including their religious and spiritual beliefs, following a specific event [42]. This scale, which Haselden (2014) adapted for Turkish, has two factors and nine items [43]. The items were rated on a six-point Likert scale ranging from 0 to 5. High scores indicate possible deterioration in fundamental assumptions about the world, including religious and spiritual beliefs. Cronbach’s alpha internal consistency coefficient of the Turkish version of the scale was 0.87.

#### Impact of Events Scale-Revısed (IES-R)

The IES-R, developed by Weiss and Marmar (1997), was used to assess the severity of trauma-related symptoms [44]. The IES-R is used to assess symptoms of post-traumatic stress disorder (PTSD) and acute stress disorder (ASD). This scale, adapted into Turkish by Çorapçıoğlu, Yargıç, Geyran, and Kocabaşoğlu (2006), has a 22-item scale that captures the main features of PTSD among individuals exposed to a specific trauma, including intrusion, avoidance, and hyperarousal. The items were rated on a six-point Likert scale ranging from 0 to 4. The scale had a minimum score of 0 and a maximum value of 88. A higher score indicated a greater impact of a traumatic event on the individual. Cronbach’s alpha internal consistency coefficients of the Turkish version of the scale of 0.94 [45].

### Statistical Analysis

SPSS version 23.0 was used to analyze the data, and PROCESS macro v4.2, an SPSS extension, was also employed to test for moderation and mediation. Frequencies, percentages, means, and standard deviations were calculated using descriptive statistics. A normality test was used to assess data distributions. Skewness and kurtosis levels between ± 2 indicate a relatively normal distribution [46]. Independent t-tests and one-way ANOVA were used to investigate the impact of sociodemographic variables and cancer-related clinical features on scale dimensions and total scale scores. Spearman’s correlation was used to analyze the correlations between the scale dimensions and the total scale scores. Cronbach’s alpha was used to assess the reliability of the scale. Listwise deletion was selected as the method to deal with missing data because of its simplicity and the presumption that the missing data occurred entirely at random. With this strategy, the analysis was limited to cases with complete data. In the present study, there were only three cases with missing data.

**Fig. 1:**
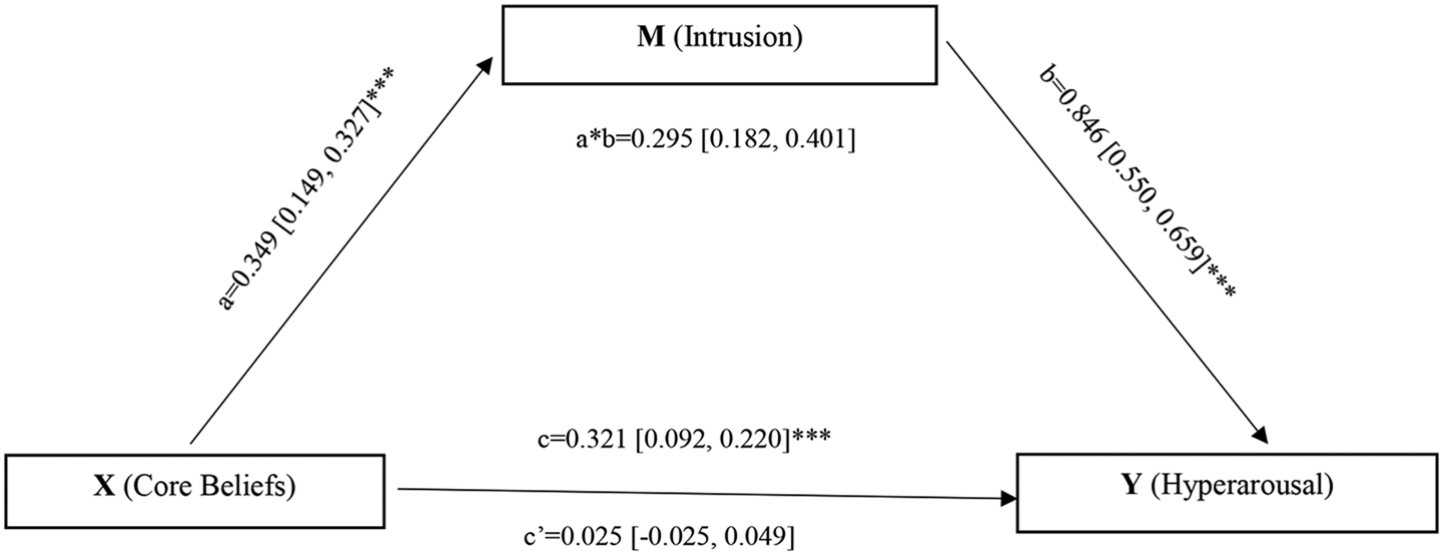
Mediation model (model 4) displaying the indirect efect of Core Beliefs on Hyperarousal via Intrusion Abbreviation: a is the direct effect of X on M; b is the direct effect of M on Y; c is the total effect; c’ is the direct effect of X on Y; a*b is the indirect effect of X on Y through M. ***p < 0.001, **p < 0.01, *p < 0.05

To evaluate the mediation hypotheses, we applied two pre-established models. As shown in Fig. 1, the simple mediation model of the association between disruption of core beliefs and hyperarousal caused by intrusion (H1) was investigated using PROCESS model 4. We employed PROCESS Model 5 (also known as a conditional process model) to test the moderated mediation hypothesis. This examined whether avoidance reduced the indirect effect of core belief disruption on hyperarousal via intrusion (H2). A 95% confidence interval and 10,000 bias-corrected bootstrap samples were used for all PROCESS tests. The importance of the interaction term was investigated by comparing the R^2^ change in the model with and without the interaction term [47]. To forecast conditional effects, the moderator’s threshold scores were 1 SD from the mean, mean, and + 1 SD from the mean. The relevance of simple regression lines for predictor conditional effects at moderator cut-off values was also evaluated. The significance level was set at *p* < 0.05.

### Ethical Considerations

Ethical Approvalwas obtained from the Ethics Committee of X University Faculty of Medicine (ethical approval number: E-60116787-020-438637; approved date: 10.10.2023), in accordance with the principles of the Declaration of Helsinki. Before starting the study, the participants were provided about the objectives of the study. Informed consent was obtained. Participants were also reminded that they could withdraw from the study if they wished and were assured that their personal data would remain confidential.

## Results

### Sample Characteristics

The demographic analysis of the study’s 204 patient participants was as follows: The following demographic breakdown applied to the study’s 204 patient participants: 55.4% of the participants were men, 87.5% were married, 66.2% had only a high school diploma, 70.6% were unemployed, 73% said they made an average income, and 75.5% came from nuclear families. Furthermore, 36.8% of the patients had lung cancer according to their diagnosis. Notably, only a small proportion of patients (34.8%) reported having family members who had the disease, and almost one-third of the patients (32.8%) received numerous treatments, including chemotherapy and surgical procedures. Years and the average deviation of 11.33 years, the participants was 59.31 years. In addition, with a standard deviation of 34.39 months, the mean time from diagnosis was 21.78 months (Table 1).

**Table 1:** Sample characteristics (*n* = 204)

				IES-*R* Total	IES-*R* subscale				Core beliefs	
Variables					Intrusion	Avoidance	Hyperarousal			
		*N*	%	Mean ± SD	Mean ± SD	Mean ± SD	Mean ± SD		Mean ± SD	
**Sex** Female Male		91	44.6	32.43 ± 11.69	12.45 ± 6.10	12.02 ± 4.88	7.96 ± 4.71		19.97 ± 9.42	
		113	55.4	25.73 ± 7.32	8.10 ± 4.80	12.18 ± 4.03	5.44 ± 3.23		16.81 ± 7.48	
*p*				*< 0.001*	*< 0.001*	*0.793*	*< 0.001*		*< 0.001*	
**Marital status** Married Single		178 26	87.3 12.7	28.61 ± 9.70 29.50 ± 12.44	10.06 ± 5.64 9.92 ± 7.09	12.10 ± 4.33 12.15 ± 5.08	6.44 ± 4.02 7.42 ± 4.98		17.97 ± 8.21 19.92 ± 10.52	
*p*				*0.676*	*0.910*	*0.960*	*0.264*		*0.279*	
**Education level** Basic education High school and higher		135 69	66.2 33.8	28.83 ± 10.09 28.50 ± 10.08	10.35 ± 5.90 9.43 ± 5.66	11.69 ± 4.65 12.92 ± 3.83	6.78 ± 4.24 6.14 ± 3.99		16.94 ± 7.68 20.73 ± 9.56	
*p*				*0.825*	*0.287*	*0.046*	*0.291*		*0.002*	
**Working status** Working Nonworking		60 144	29.4 70.6	27.80 ± 8.54 29.11 ± 10.64	9.71 ± 5.43 10.18 ± 5.99	12.53 ± 4.02 11.93 ± 4.58	5.55 ± 3.16 6.99 ± 4.45		18.18 ± 9.00 18.24 ± 8.36	
*p*				*0.398*	*0.606*	*0.382*	*0.010*		*0.94*	
**Family type** Extended family Nuclear family		50 154	24.5 75.5	30.04 ± 10.93 28.29 ± 9.76	10.42 ± 6.06 9.92 ± 5.75	12.78 ± 3.90 11.89 ± 4.57	6.84 ± 4.75 6.48 ± 3.96		16.46 ± 8.48 18.79 ± 8.50	
*p*				*0.289*	*0.601*	*0.221*	*0.597*		*0.092*	
**Income status** Income less than expenses^1^ Income equal to expenses^2^ Income more than expenses^3^		26 149 29	12.7 73 14.2	30.73 ± 11.62 29.10 ± 10.10 28.72 ± 10.06	11.76 ± 6.69 10.14 ± 5.72 7.54 ± 4.78	11.23 ± 3.45 12.22 ± 4.63 12.33 ± 4.07	7.73 ± 4.69 6.73 ± 4.15 4.25 ± 2.59		16.88 ± 8.26 18.48 ± 8.60 18.00 ± 8.57	
*p* *Bonferroni*				*0.043* *1 > 3*	*0.033* *1 > 3*	*0.552*	*0.007* *1 > 3; 2 > 3*		*0.671*	
**Family history of cancer** Yes No		71 133	34.8 65.2	30.39 ± 9.96 27.83 ± 10.04	11.04 ± 6.24 9.51 ± 5.53	12.21 ± 4.36 12.06 ± 4.47	7.14 ± 4.37 6.26 ± 4.02		18.04 ± 8.75 18.32 ± 8.44	
*p*				*0.084*	*0.074*	*0.817*	*0.152*		*0.823*	
***Type of cancer***										
Lung Cancer^1^		75	36.8	27.61 ± 10.32	8.92 ± 5.62	12.54 ± 4.66	6.14 ± 4.03		17.93 ± 8.39	
Breast Cancer^2^		52	25.5	30.55 ± 10.18	11.84 ± 6.06	11.38 ± 4.83	7.32 ± 4.124		19.61 ± 9.82	
Colon Cancer^3^		30	14.7	27.50 ± 8.20	9.40 ± 5.46	12.00 ± 4.12	6.10 ± 3.80		17.90 ± 7.64	
Utherine Cancer^4^		15	7.4	32.13 ± 11.81	12.20 ± 6.77	12.26 ± 3.49	7.66 ± 5.28		19.60 ± 7.87	
Other^5^		32	15.7	27.90 ± 9.82	9.34 ± 5.06	12.31 ± 3.86	6.25 ± 4.25		16.31 ± 7.70	
*p* *Bonferroni*				*0.287*	*0.029* *2 > 1*	*0.697*	*0.394*		*0.480*	
**Current treatment**										
Chemotherapy		77	37.7	28.31 ± 11.10	9.94 ± 5.67	11.70 ± 4.41	6.66 ± 4.23		18.37 ± 8.41	
Chemotherapy + Radiotherapy		33	16.2	29.36 ± 7.74	10.63 ± 5.46	11.78 ± 4.09	6.93 ± 3.56		19.09 ± 7.24	
Chemotherapy + Surgical		67	32.8	29.35 ± 10.07	10.43 ± 6.29	12.46 ± 4.88	6.46 ± 4.35		18.70 ± 9.27	
Chemotherapy + Radiotherapy + Surgical		27	13.2	27.55 ± 9.81	8.62 ± 5.51	12.81 ± 3.64	6.11 ± 4.31		15.55 ± 8.35	
*p*				0.830	0.525	0.588	0.882		0.362	
	**Min.-Max. Mean ± SD**									
	**Age** 19–85 59.59 ± 11.16									
	**Patients” time since diagnosis (month**) 2-240 21.78 ± 34.39									

### Comparison of the Mean Scores of Scales According to Socio-Demographic Characteristics of Participants

Table 1 shows how participants’ sociodemographic variables affect the IES-R overall score, its subscales, and the scores for basic distraction beliefs. With regard to gender, women had higher IES-R total scores and its subscales intrusion and hyperarousal, as well as distraction scores in core beliefs, than males, and this difference was statistically significant (*p* < 0.001). When education level was considered, high school graduates and above had higher avoidance subscale (*p* = 0.046) and distraction in core belief scores (*p* = 0.002) than basic education graduates, and this difference was statistically significant. When considering working status, it was found that the mean hyperarousal subscale score of those who were not working was significantly higher than those who were working (*p* = 0.010). The IES-R total score (*p* = 0.043) and its subscale intrusion (*p* = 0.033) and hyperarousal mean scores (*p* = 0.007) were significantly higher in situations where income was less than expenses than in situations where income was greater than expenses, according to an analysis of income status. Furthermore, the mean hyperarousal scores were higher in situations where income was equal to expenses than in situations where income was higher than expenses. The mean intrusion subscale score of breast cancer participants was significantly higher than that of lung cancer participants (*p* = 0.029). The other groups showed no statistically significant differences (*p* > 0.05).

### Descriptive Analyses and Correlations

Table 2 provides descriptive data, correlation analysis outcomes, and internal reliability estimates for the study variables. According to the reliability analysis, the internal reliability estimates for all the research variables for the current sample ranged from 0.695 to 0.903. The study’s variables’ skewness and kurtosis values show that they fall within the bounds of a normal distribution. Additional analyses indicated that distraction in core beliefs was positive and significantly correlated with intrusion (*r* = 0.349, *p* < 0.01). And hyperarousal (*r* = 0.321, *p* < 0.01) yet had an insignificant correlation with avoidance. Furthermore, while there was a low negative association between intrusion and avoidance (*r*=-0.175, *p* < 0.05), there was a moderate positive association between hyperarousal and intrusion (*r* = 0.855, *p* < 0.01).

**Table 2:** Descriptive statistics and correlations for study variables

Variables	Mean ± SD	Skewness	Kurtosis	Min-Max.	α	1.		2.	3.	4.	
1. Core beliefs	18.22 ± 8.53	0.471	-0.340	1–42	0.903	1					
2. Intrusion	10.04 ± 5.82	0.788	0.399	0–30	0.873	0.349**	1				
3. Avoidance	12.11 ± 4.42	0.430	0.691	2–28	0.695	0.074	− 0.175*1				
4. Hyperarousal	6.56 ± 4.16	1.316	1.446	0–21	0.799	0.321**	0.855** − 0.052				1

**p < 0.01; *p < 0.05

### Mediation Analyses

Table 3 and Fig. 1 show the findings of the mediation study examining the indirect effect of distraction core beliefs on hyperarousal through intrusion. As there was only one mediator variable, Model 4 was used to perform a simple mediation analysis. There is a strong mediation impact of distraction core beliefs on hyperarousal through intrusion, as evidenced by the bias-adjusted bootstrap confidence interval for the indirect effect (a*b) not crossing zero (effect = 0.29, BootSE = 0.056, BootLLCI = 0.182, BootULCI = 0.401). This finding supports Hypothesis 1. Paths a, b and c were statistically significant (Fig. 1). The direct effect (c’) of distraction in core beliefs on hyperarousal was no longer significant (*p* = 0.512) indicating that intrusion fully mediated the relationship between distraction in core beliefs and hyperarousal.

**Table 3:** Results of Regression-Based mediation model showing the effect of core beliefs on hyperarousal via intrusion

Effect (path)	Coeff.	SE	t	*p*	LLCI	ULCI	*R* ^2^
Core beliefs- Intrusion *(a)*	0.349	0.045	5.295	*0.000*	0.149	0.327	%12
Intrusion -Hyperarousal *(b)*	0.846	0.027	21.732	*0.000*	0.550	0.659	%73
Core beliefs – Hyperarousal (Direct effect) *(c’)*	0.025	0.019	0.655	*0.512*	-0.025	0.049	
*Total Effect* (c)	0.321	0.032	4.818	*0.000*	0.092	0.220	
	***Effect***	***Boot SE***	***BootLLCI***	***BootULCI***			
*Bias corrected bootstrap test of standardized indirect effect*							
Core beliefs - Intrusion - Hyperarousal (a*b)	0.295*	0.056	0.182	0.401			

Abbreviations: Coeff: Standardized regression coefficient; LLCI: Lower level of the 95% confidence interval; SE: Standard error; ULCI: Upper level of the 95% confidence interval.*Significant effect, Model 4

In this case, the mediator variable was said to have a significant effect on the dependent variable. Distruption in basic beliefs accounted for 12% of the participants’ intrusion scores [R^2^ = 0.12, F (1, 202) = 28.04, *p* < 0.001]. 73% of hyperarousal scores were explained by distraction in basic beliefs and intrusion [R^2^ = 0.73, F (2, 201) = 274.83, *p* < 0.001].

### Moderated Mediation Analyses

**Fig. 2:**
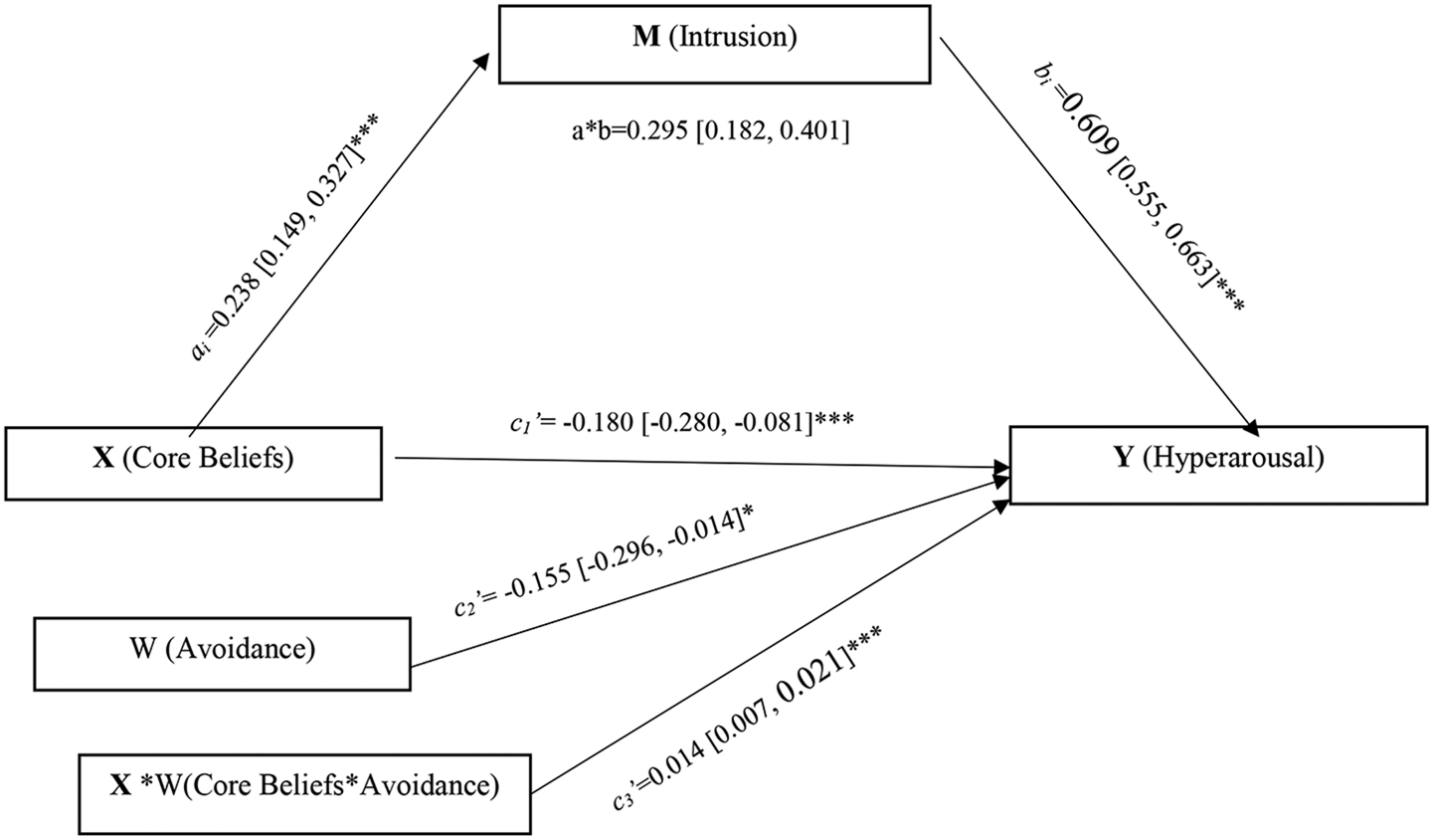
Moderated mediation (model 5)***p < 0.001, **p < 0.01, *p < 0.05

The PROCESS macro was used to evaluate the moderated mediation model using Model 5, which is suggested in Hypothesis 2, and states that avoidance moderates the mediation of intrusion between distruption in core beliefs and hyperarousal. Table 4 summarizes the results of the moderated mediation model (Fig. 2). While paths a_i_, b_i_, c_2_’ and c_3_’ were statistically significant, path c_1_’ was not. The interaction path (c_3_’) was significant, showing that the relationship between distraction in core beliefs and hyperarousal was moderated by avoidance (β = 0.014, *p* < 0.001). According to the analysis results, the model’s explanatory power (R^2^) was found to be 0.12 for the “Intrusion” variable and 0.76 for the “Hyperarousal” variable. Additionally, according to the F-test results, the model was found to be statistically significant (F (1, 202) = 28.040, *p* < 0.001; F (4, 199) = 157.919, *p* < 0.001, Table 4).

**Table 4:** Ordinary least squares regression coefficients for conditional indirect effect of core beliefs on hyperarousal through intrusion, with avoidance as moderator

Outcome	Intrusion							Hyperarousal									
Predictor	Path	Coeff.	SE	t	*p*	LLCI	ULCI	Path	Coeff.		SE		t	*p*	LLCI		ULCI
Constant		5.701	0.905	6.298	0.000	3.916	7.486		2.445		1.015		2.409	*0.016*	0.443		4.448
Core Beliefs	*a*_*i*_	0.238	0.045	5.295	0.000	0.149	0.327	*c*_*1*_ *’*	-0.180		0.050		-3.580	*0.000*	-0.280		-0.081
Intrusion		-	-	-	-			*b*_*i*_	0.609		0.027		22.367	*0.000*	0.555		0.663
Avoidance		-	-	-	-			*c*_*2*_ *’*	-0.155		0.071		-2.172	*0.031*	-0.296		-0.014
Core Beliefs* Avoidance		-	-	-	-			*c*_*3*_ *’*	0.014		0.003		3.945	*0.000*	0.007		0.021
*R*^*2*^ *= 0.12* *F(1*,*202 ) = 28.040*, *p* = 0.000								*R*^*2*^ *= 0.76* *F(4*,* 199) = 157.919*, *p* = 0.000									
*Conditional direct effects of core beliefs on hyperarousal*																	
*Avoidance*						***Effect***		***SE***		***t***		***p***		***LLCI***		***ULCI***	
*M-1 SD*						-0.067		0.025		-2.598		0.010		-0.118		-0.016	
*M (0.00)*						-0.010		0.018		-0.546		0.585		-0.047		0.026	
*M + 1 SD*						0.046		0.021		2.212		0.028		0.005		0.088	
						***Effect***		***BootSE BootLLCI***						***BootULCI***			
*Indirect effect of core beliefs on hyperarousal*						0.145*		0.030		0.087				0.204			

Abbreviations: BootLLCI: Lower level of the 95% confidence interval; BootULCI: Upper level of the 95% confidence interval; Coeff.: Unstandardized regression coefficients; R^2^: Coefficient of determination; SE: standard error. *p* < 0.001*Significant effect, Model 5

**Fig. 3:**
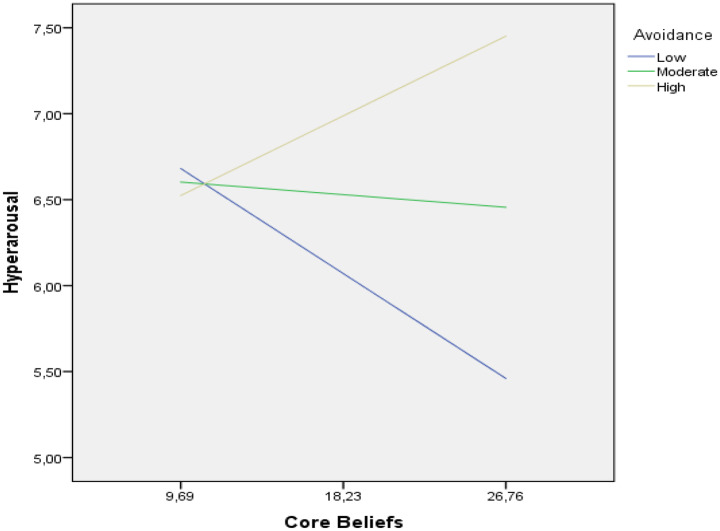
The simple slope indicating the moderation effects

The avoidance score was divided based on M, M + SD, and M-SD, resulting in three distinct levels: medium, low, and high, respectively. Table 4; Fig. 3 show the conditional direct effects of distraction in basic beliefs on hyperarousal at different levels of avoidance. It was found that under a moderate level of avoidance, the direct effect of distraction in basic beliefs on hyperarousal was not significant, and under high and low levels of avoidance, the direct effect of distraction in basic beliefs on hyperarousal was significant. The confidence intervals of the 95% CI were [-0.118,-0.016], [-0.047, 0.026], and [0.005, 0.088] at − 1, 0, and + 1 SD, respectively. In this case, when avoidance behavior is at a high level, hyperarousal symptoms increase as the distraction in basic beliefs increases. When the participants’ avoidance behavior was low, the disturbance in core beliefs had a lower influence on hyperarousal symptoms (Fig. 3). The estimated moderating effect of avoidance on indirect effects was 0.145. The bias-corrected 95% confidence interval was [0.087, 0.204]. Therefore, the moderating effect of avoidance on the indirect effect is also significant. Avoidance significantly moderated the mediating relationship between distraction in basic beliefs, hyperarousal, and intrusion (Table 4).

## Disscussion

This study set out to examine how disruptions in core beliefs relate to post-traumatic stress disorder (PTSD) symptoms in cancer patients, with a particular focus on potential mediators (intrusion) and moderators (avoidance behavior). First, we assessed the impact of sociodemographic variables on PTSD symptoms to establish a contextual backdrop. We found significant associations between gender, income level, employment status, and educational attainment and overall PTSD symptom severity. Although Cordova et al. (1995) reported similar links, George (2016) found no such relationships, and Shand et al. (2015) highlighted inconsistencies, suggesting that demographic influences on cancer-related PTSD warrant further investigation [20, 48, 49]. Additionally, the present study identified heightened levels of hyperarousal symptoms in the sample. This finding aligns with Zhou et al. (2019) but contrasts with earlier reports such as Steenkamp et al. (2012) [50, 51], thereby underscoring the variability in trauma responses across cancer populations and reinforcing the need to explore underlying cognitive mechanisms.

This study contributes to the theoretical understanding of PTSD by demonstrating that disruptions in core beliefs are positively associated with both intrusion and hyperarousal. This relationship is consistent with cognitive frameworks that emphasize how traumatic experiences can destabilize foundational assumptions about the self and the world [34–54]. Cancer, in particular, imposes persistent emotional stress, which may reinforce negative belief systems and exacerbate psychological symptoms [19]. Horowitz’s stress-response theory suggests that when life events contradict an individual’s internal schemas, adaptive mechanisms may be overwhelmed, leading to persistent symptoms such as intrusion and hyperarousal [38]. More recent work has corroborated these patterns using moderated mediation models [56].

A central finding of this study is that the association between core-belief disruption and hyperarousal operates indirectly through intrusion. This full mediation implies that disrupted beliefs primarily influence hyperarousal by increasing the frequency or intensity of intrusive thoughts. Theoretical support for this mechanism comes from Janoff-Bulman (1989), who proposed that traumatic stress arises when core assumptions collapse, and from Horowitz’s conceptualization of active memory and completeness tendency, which explain how unprocessed traumatic information can remain accessible and disruptive in conscious awareness [38, 57, 58, 59]. Similarly, Foa and colleagues’ fear-network theory describes how trauma-related cues may activate maladaptive cognitive structures, resulting in both intrusion and heightened arousal [37, 60, 61, 62].

### Avoidance Behavior as a Key Moderator

In addition to this mediating pathway, our analyses identified avoidance behavior as a significant moderator. The moderated mediation analysis (Model 5) provided additional nuance, revealing that avoidance behavior significantly alters the direct pathway from core-belief disruption to hyperarousal. Notably, while core-belief disruption initially showed a nonsignificant direct effect in the simple mediation model, the inclusion of avoidance as a moderator unveiled a significant interaction (b = 0.014, *p* < 0.001). These findings underscore the context-dependent nature of trauma processing, whereby avoidance amplifies the physiological consequences of disrupted beliefs. Avoidance significantly shaped the direction and strength of the direct effect of core-belief disruption on hyperarousal, functioning as a context-dependent amplifier or buffer. The interaction between belief disruption and avoidance behavior was statistically significant (c₃′ = 0.014, *p* < 0.001), indicating that the direct effect of core-belief disruption on hyperarousal varied depending on the level of avoidance. Specifically, among participants with high levels of avoidance (1 SD above the mean), core-belief disruption was positively and significantly associated with hyperarousal (effect = 0.046, *p* = 0.028), indicating that elevated avoidance intensifies the adverse impact of disrupted beliefs. In contrast, among individuals with low avoidance (1 SD below the mean), the association was negative and statistically significant (effect = − 0.067, *p* = 0.010), suggesting a potential buffering or adaptive effect when individuals are more open to confronting distressing thoughts. Notably, at moderate levels of avoidance (mean level), the relationship between core-belief disruption and hyperarousal was nonsignificant (effect = − 0.010, *p* = 0.585), reflecting a neutralization of effects at average levels of avoidance.

This pattern is consistent with cognitive-behavioral accounts of PTSD. Avoidance, while serving as a short-term coping strategy, can hinder emotional and cognitive processing of trauma-related material, thereby maintaining or exacerbating distress [63, 64]. In contrast, lower levels of avoidance may facilitate engagement with traumatic content, promoting habituation and restructuring through repeated exposure [65–67]. These findings suggest that avoidance is not merely a correlational variable but a dynamic factor that alters the directionality and magnitude of trauma-related psychological outcomes. The distinct effects at low, moderate, and high avoidance levels underscore the need to consider avoidance as a spectrum. At high avoidance, disrupted beliefs exacerbate arousal symptoms, indicating the need for exposure-based restructuring. At low avoidance, disrupted beliefs may correlate with reduced arousal, suggesting greater emotional processing. At moderate avoidance, the absence of a significant effect implies a regulatory plateau where therapeutic gains may depend on enhancing insight or emotional tolerance. These differentiated patterns highlight that interventions should be flexibly adapted to patients’ avoidance tendencies rather than uniformly applied.

From a clinical standpoint, these results highlight the importance of evaluating and targeting avoidance behaviors in PTSD interventions. Individuals exhibiting high avoidance may particularly benefit from structured exposure-based treatments that support the integration of traumatic memories and the revision of maladaptive core beliefs. Future research may consider whether combining exposure therapy with interventions that directly address belief systems enhances therapeutic outcomes in cancer populations.

In sum, this study offers empirical support for an integrative model in which core-belief disruptions are linked to PTSD symptomatology through intrusion, and where the strength of this relationship is moderated by avoidance. By clarifying the roles of both mediation and moderation, these findings contribute to the refinement of trauma theory and suggest promising avenues for tailored psychological interventions in oncology settings. Taken together, these findings advance a cohesive explanatory model that integrates cognitive disruption, trauma processing, and behavioral regulation in the context of cancer-related PTSD.

### Study Limitations

This study has several methodological limitations that should be carefully considered. First, although the sample size was adequate for the statistical analyses conducted, the use of a single-center, non-probability sampling method and a cross-sectional design limits both the generalizability of the findings and the ability to infer causality. Future research employing multi-center and longitudinal designs may offer stronger evidence for causal mechanisms.

Second, the data were collected through self-report instruments, which may introduce cognitive biases such as recall errors. Additionally, as PTSD-related symptoms were assessed solely through subjective evaluations, the absence of clinical observation may have limited the comprehensiveness of the symptom assessment. Third, while this study focused on the mediating role of intrusion and the moderating role of avoidance behavior, other potentially relevant variables—such as coping styles, illness perception, prior trauma exposure, and psychological resilience—were not included in the model. Excluding these covariates may have restricted a more holistic understanding of the mechanisms linking core belief disruption and PTSD symptoms. Finally, as the instruments used were adapted into Turkish, cultural nuances may have influenced how participants interpreted constructs like “core belief disruption.” This may, in turn, affect the cross-cultural validity of the findings. Considering these limitations, future research should aim to replicate and expand upon the current results by employing more diverse samples, theory-driven models, and methodologically rigorous designs.

## Conclusion and Implications for Practice

This study provides critical insights into how disruptions in core beliefs are associated with posttraumatic stress symptoms, particularly hyperarousal, in individuals diagnosed with cancer. The findings demonstrate that this association is fully mediated by intrusion and conditionally moderated by avoidance behavior. Notably, the relationship between core belief disruption and hyperarousal was found to be significant at both low and high levels of avoidance, while it was not significant at moderate levels. This nonlinear pattern underscores the necessity of evaluating trauma responses within the broader context of individual coping dynamics and cognitive vulnerability.

From a clinical perspective, these results suggest that psychosocial interventions should not focus solely on observable symptoms but must also address the underlying cognitive schemas and coping tendencies that sustain those symptoms. Disruptions in fundamental assumptions about the self, the world, and the future may compromise psychological adjustment, particularly when coupled with maladaptive patterns of avoidance or excessive emotional exposure. According to, therapeutic processes should prioritize the evaluation and restructuring of disrupted belief systems. Structured trauma therapies (e.g., written exposure, guided imagery) may help patients articulate, process, and integrate distressing cancer-related experiences. This study demonstrated that the effect of core belief disruption on hyperarousal significantly varies depending on the level of avoidance. This finding underscores the necessity of adapting interventions to individuals’ avoidance profiles.

In sum, this study contributes to a deeper understanding of trauma-related cognitive mechanisms in cancer patients and highlights the importance of individualized, cognitively informed, and trauma-sensitive interventions. Future research may build upon these findings through longitudinal and intervention-based designs to further validate and extend their clinical applicability.

## Electronic Supplementary Material

Below is the link to the electronic supplementary material.

Supplementary Material 1

## Data Availability

The datasets generated during and/or analysed during the current study are available from the corresponding author on reasonable request.

## References

[1] Vineis P, Wild CP. Global cancer patterns: causes and prevention. Lancet. 2014;383(9916):549–57. 24351322 10.1016/S0140-6736(13)62224-2

[2] Zhang S, Sun K, Zheng R, Zeng H, Wang S, Chen R, He J. Cancer incidence and mortality in china, 2015. J Natl Cancer Cent. 2021;1(1):2–11. 39036787 10.1016/j.jncc.2020.12.001 PMC11256613

[3] Bray F, Ferlay J, Soerjomataram I, Siegel RL, Torre LA, Jemal A. Global cancer statistics 2018: GLOBOCAN estimates of incidence and mortality worldwide for 36 cancers in 185 countries. Cancer J Clin. 2018;68(6):394–424. 10.3322/caac.21492 30207593

[4] Stein KD, Syrjala KL, Andrykowski MA. Physical and psychological long-term and late effects of cancer. Cancer. 2008;112(S11):2577–92. 18428205 10.1002/cncr.23448 PMC7047657

[5] Al-Saadi L, Chan M, Al-Azri M. Prevalence of anxiety, depression, and Post-Traumatic stress disorder among children and adolescents with cancer: A systematic review and Meta-Analysis. J Pediatr Hematology/Oncology Nurs. 2022;39:114–31. 10.1177/27527530211056001. 10.1177/27527530211056001 35722683

[6] Low C, Loke S, Pang G, Sim B, Yang V. Psychological outcomes in patients with rare cancers: a systematic review and meta-analysis. eClinicalMedicine. 2024;72. 10.1016/j.eclinm.2024.102631. 10.1016/j.eclinm.2024.102631 PMC11079476 38726223

[7] Huda N, Shaw M, Chang H. Psychological distress among patients with advanced Cancer. Cancer Nurs. 2021;45:E487–503. 10.1097/NCC.0000000000000940. 10.1097/NCC.0000000000000940 33813528

[8] Fortin J, LeBlanc M, Elgbeili G, Cordova M, Marin M, Brunet A. The mental health impacts of receiving a breast cancer diagnosis: A meta-analysis. Br J Cancer. 2021;125:1582–92. 10.1038/s41416-021-01542-3. 34482373 10.1038/s41416-021-01542-3 PMC8608836

[9] Oh H, Son C. The risk of psychological stress on cancer recurrence: a systematic review. Cancers. 2021;13. 10.3390/cancers13225816.). 10.3390/cancers13225816 PMC8616395 34830968

[10] Cordova MJ, Andrykowski MA, Kenady DE, McGrath PC, Sloan DA, Redd WH. Frequency and correlates of posttraumatic-stress-disorder-like symptoms after treatment for breast cancer. J Consult Clin Psychol. 1995;63(6):981. 8543720 10.1037//0022-006x.63.6.981

[11] Mystakidou K, Parpa E, Tsilika E, Panagiotou I, Roumeliotou A, Galanos A, Gouliamos A. Traumatic experiences of patients with advanced cancer. J Loss Trauma. 2012;17(2):125–36.

[12] Alter CL, Pelcovitz D, Axelrod A, Goldenberg B, Harris H, Meyers B, Kaplan S. Identification of PTSD in cancer survivors. Psychosomatics. 1996;37(2):137–43. 8742542 10.1016/S0033-3182(96)71580-3

[13] Leano A, Korman MB, Goldberg L, Ellis J. Are we missing PTSD in our patients with cancer? Part I. Can Oncol Nurs J. 2019;29(2):141. 31148714 PMC6516338

[14] American Psychiatric Association. Diagnostic and Statistical Manual of Mental Disorders (4th edn). Author: Washington, DC, 1994.

[15] Marziliano A, Tuman M, Moyer A. The relationship between post-traumatic stress and post‐traumatic growth in cancer patients and survivors: A systematic review and meta‐analysis. Psycho‐Oncology. 2020;29(4):604–16. 31834657 10.1002/pon.5314

[16] Unseld M, Krammer K, Lubowitzki S, Jachs M, Baumann L, Vyssoki B, Gaiger A. Screening for post-traumatic stress disorders in 1017 cancer patients and correlation with anxiety, depression, and distress. Psycho‐Oncology. 2019;28(12):2382–8. 31679172 10.1002/pon.5239 PMC6916606

[17] Bruce M. A systematic and conceptual review of posttraumatic stress in childhood cancer survivors and their parents. Clin Psychol Rev. 2006;26(3):233–56. 16412542 10.1016/j.cpr.2005.10.002

[18] Abbey G, Thompson S, Hickish T, Heathcote D. A meta-analysis of prevalence rates and moderating factors for cancer-related post-traumatic stress disorder. Psycho-oncology. 2014;24:371–81. 10.1002/pon.3654. 25146298 10.1002/pon.3654 PMC4409098

[19] Swartzman S, Booth J, Munro A, Sani F. Posttraumatic stress disorder after cancer diagnosis in adults: a meta-analysis. Depress Anxiety. 2017;34. 10.1002/da.22542. 10.1002/da.22542 27466972

[20] Arnaboldi P, Riva S, Crico C, Pravettoni G. A systematic literature review exploring the prevalence of post-traumatic stress disorder and the role played by stress and traumatic stress in breast cancer diagnosis and trajectory. Breast Cancer: Targets Therapy. 2017;9:473–85. 10.2147/BCTT.S111101. 28740430 10.2147/BCTT.S111101 PMC5505536

[21] Cordova MJ, Riba MB, Spiegel D. Post-traumatic stress disorder and cancer. Lancet Psychiatry. 2017;4(4):330–8. 28109647 10.1016/S2215-0366(17)30014-7 PMC5676567

[22] American Psychiatric Association. Diagnostic and Statistical Manual of Mental Disorders (5th edn). Author: Washington, DC, 2013.

[23] Doolittle M, DuHamel K. Posttraumatic stress disorder associated with Cancer diagnosis and treatment. Psycho-oncology. 2021. 10.1093/med/9780190097653.003.0047.

[24] Franks H, Roesch S. Appraisals and coping in people living with cancer: a meta-analysis. Psycho‐Oncology. 2006;15. 10.1002/pon.1043. 10.1002/pon.1043 16602072

[25] Yang X, Wu X, Gao M, Wang W, Quan L, Zhou X. Heterogeneous patterns of posttraumatic stress symptoms and depression in cancer patients. J Affect Disord. 2020;273:203–9. 10.1016/j.jad.2020.04.033. 32421604 10.1016/j.jad.2020.04.033

[26] Caldas S, Fondren A, Batley P, Contractor A. Longitudinal relationships among posttraumatic stress disorder symptom clusters in response to positive memory processing. J Behav Ther Exp Psychiatry. 2022;76:101752. 10.1016/j.jbtep.2022.101752. 35738684 10.1016/j.jbtep.2022.101752

[27] Fardell J, Thewes B, Turner J, Gilchrist J, Sharpe L, Smith A, Girgis A, Butow P. Fear of cancer recurrence: a theoretical review and novel cognitive processing formulation. J Cancer Surviv. 2016;10:663–73. 10.1007/s11764-015-0512-5. 26782171 10.1007/s11764-015-0512-5

[28] Russell G, Lightman S. The human stress response. Nat Reviews Endocrinol. 2019;1–10. 10.1038/s41574-019-0228-0. 10.1038/s41574-019-0228-0 31249398

[29] Kangas M. DSM-5 trauma and stress-related disorders: implications for screening for cancer-related stress. Front Psychiat. 2013. 10.3389/fpsyt.2013.00122. 10.3389/fpsyt.2013.00122 PMC3788331 24106482

[30] Rustad JK, David D, Currier MB. Cancer and post-traumatic stress disorder: diagnosis, pathogenesis and treatment considerations. Palliat Support Care. 2012;10(3):213–23. 10.1017/S1478951511000897. 22436138 10.1017/S1478951511000897

[31] Anderson D, Jones V. Psychological interventions for cancer-related post-traumatic stress disorder: narrative review. BJPsych Bull. 2023;48:100–9. 10.1192/bjb.2023.42. 10.1192/bjb.2023.42 PMC10985725 37288666

[32] Nenova M, Morris L, Paul L, Li Y, Applebaum A, DuHamel K. Psychosocial interventions with Cognitive-Behavioral components for the treatment of Cancer-Related traumatic stress symptoms: A review of randomized controlled trials. J Cogn Psychother. 2013;27:258–84. 10.1891/0889-8391.27.3.258. 32759144 10.1891/0889-8391.27.3.258 PMC11056102

[33] Catlin G, Epstein S. Unforgettable experiences: the relation of life events to basic beliefs about self and world. Soc Cogn. 1992;10(2):189–209.

[34] Silver RL, Wortman CB. Coping with undesirable life events. In: Garber J, Seligman MEP, editors. Human helplessness. New York: Academic; 1980.

[35] VandenBos GR, Bryant BK, editors. Cataclysms, crises, and catastrophes: psychology in action. Washington, DC: American Psychological Association; 1987.

[36] Fletcher K. (1988). Belief systems, exposure to stress, and post-traumatic stress disorder in Vietnam veterans. Doctoral Dissertation, University of Massachusetts at Amherst.

[37] Epstein S. The self-concept, the traumatic neurosis, and the structure of personality. In: Ozer D, Healy JM Jr, Stewart AJ, editors. Perspectives on personality. Volume 3. London: Jessica Kingsley; 1991.

[38] Foa EB, Zinbarg R, Rothbaum BO. Uncontrollability and unpredictability in posttraumatic stress disorder: an animal model. Psychol Bull. 1992;112:218–38. 1454893 10.1037/0033-2909.112.2.218

[39] Horowitz MJ. Stress-response syndromes: A review of posttraumatic and adjustment disorders. Psychiatric Serv. 1986;37(3):241–9. 10.1176/ps.37.3.241 3957267

[40] Janoff-Bulman R. Shattered assumptions: towards a new psychology of trauma. New York, NY: Free; 1992.

[41] Ogden P, Pain C, Fisher J. A sensorimotor approach to the treatment of trauma and dissociation. Psychiatric Clin. 2006;29(1):263–79. 10.1016/j.psc.2005.10.012 16530597

[42] Cann A, Calhoun LG, Tedeschi RG, Kilmer RP, Gil-Rivas V, Vishnevsky T, Danhauer SC. The core beliefs inventory: A brief measure of disruption in the assumptive world. Anxiety Stress Coping: Int J. 2010;23:19–34. 10.1080/10615800802573013. 10.1080/10615800802573013 19326274

[43] Haselden M. Üniversite öğrencilerinde Travma Sonrası Büyümeyi Yordayan çeşitli Değişkenlerin Türk ve Amerikan kültürlerinde incelenmesi. Bir model önerisi;. 2014.

[44] Weiss D, Marmar C. The impact of event Scale - Revised. In: Wilson J, Keane T, editors. Assessing psychological traumaand PTSD. New York: Guilford; 1997.

[45] Çorapçıoğlu A, Yargıç İ, Geyran P, Kocabaşoğlu N. (2006). Validity and reliability of Turkish version of impact of event scale-revised (IES-R). In Yeni Symposium 44(1):14-22.

[46] Bachman LF. Statistical analyses for language assessment book. Cambridge University Press. 2004.

[47] Hayes AF, Montoya AK, Rockwood NJ. The analysis of mechanisms and their contingencies: PROCESS versus structural equation modeling. Australasian Mark J. 2017;25(1):76–81.

[48] George LS, Park CL, Chaudoir SR. Examining the relationship between trauma centrality and posttraumatic stress disorder symptoms: A moderated mediation approach. Traumatology. 2016;22(2):85. 27458331 10.1037/trm0000063 PMC4957691

[49] Shand LK, Cowlishaw S, Brooker JE, Burney S, Ricciardelli LA. Correlates of post-traumatic stress symptoms and growth in cancer patients: A systematic review and meta‐analysis. Psycho‐oncology. 2015;24(6):624–34. 25393527 10.1002/pon.3719

[50] Zhou X, Gao M, Wang W, Wu X. Patterns of posttraumatic stress disorder symptoms among cancer patients: a latent profile analysis. J Psychosom Res. 2019;125:109788. 31421322 10.1016/j.jpsychores.2019.109788

[51] Steenkamp MM, Nickerson A, Maguen S, Dickstein BD, Nash WP, Litz BT. Latent classes of PTSD symptoms in Vietnam veterans. Behav Modif. 2012;36(6):857–74. 22798638 10.1177/0145445512450908

[52] Halldórsdóttir S, Hamrin E. Experiencing existential changes: the lived experience of having cancer. Cancer Nurs. 1996;19(1):29–36. 8904384 10.1097/00002820-199602000-00004

[53] Lagerdahl AS, Moynihan M, Stollery B. An exploration of the existential experiences of patients following curative treatment for cancer: reflections from a UK sample. J Psychosoc Oncol. 2014;32(5):555–75. 25045924 10.1080/07347332.2014.936647

[54] Simard S, Savard J. Fear of Cancer recurrence inventory: development and initial validation of a multidimensional measure of fear of cancer recurrence. Support Care Cancer. 2009;17:241–51. 18414902 10.1007/s00520-008-0444-y

[55] Mellon S, Kershaw TS, Northouse LL, Freeman-Gibb L. A family‐based model to predict fear of recurrence for cancer survivors and their caregivers. Psycho‐Oncology: J Psychol Social Behav Dimensions Cancer. 2007;16(3):214–23. 10.1002/pon.1074 16906624

[56] Chukwuorji JBC, Ifeagwazi CM, Eze JE. Event centrality influences posttraumatic stress disorder symptoms via core beliefs in internally displaced older adults. Aging Ment Health. 2019;23(1):113–21. 29099623 10.1080/13607863.2017.1396580

[57] Janoff-Bulman R. Assumptive worlds and the stress of traumatic events: applications of the schema construct. Soc Cogn. 1989;7(2):113–36.

[58] Janoff-Bulman R, Frieze IH. A theoretical perspective for Understanding reactions to victimization. J Soc Issues. 1983;39(2):1–17.

[59] Horowitz MJ. Stress response syndromes. 3rd ed. Northvale, NJ: Aronson; 1997.

[60] Foa EB, Kozak MJ. Emotional processing of fear: exposure to corrective information. Psychol Bull. 1986;99(1):20–35. 2871574

[61] Foa EB, Riggs DS. Post-traumatic stress disorder in rape victims. In: Oldham J, Riba MB, Tasman A, editors. American psychiatric press review of psychiatry (\o\. 12. Washington, DC: American Psychiatric; 1993. pp. 273–303.

[62] Foa EB, Steketee G, Rothbaum BO. Behavioral/ cognitive conceptualization of post-traumatic stress disorder. Behav Ther. 1989;20:155–76.

[63] Tull MT, Gratz KL, Salters K, Roemer L. The role of experiential avoidance in posttraumatic stress symptoms and symptoms of depression, anxiety, and somatization. J Nerv Ment Dis. 2004;192(11):754–61. 15505519 10.1097/01.nmd.0000144694.30121.89

[64] Briere J, Hodges M, Godbout N. Traumatic stress, affect dysregulation, and dysfunctional avoidance: A structural equation model. J Trauma Stress. 2010;23(6):767–74. 21171138 10.1002/jts.20578

[65] McLean CP, Levy HC, Miller ML, Tolin DF. Exposure therapy for PTSD: A meta-analysis. Clin Psychol Rev. 2022;91:102115. 34954460 10.1016/j.cpr.2021.102115

[66] Wolpe J. The practice of behavior therapy. New York: Pergamon; 1969.

[67] Antony MM, Rowa K. Psychological treatments for social phobia. Can J Psychiatry. 2005;50(6):308–15. 15999944 10.1177/070674370505000603

