# Supplementary material for: The Impact of Core Belief Disruption on PTSD Symptoms in Cancer Patients: The Mediating and Moderating Roles of Intrusion and Avoidance Behavior
Source: Psychiatr Q. 2025 Jul 1;96(3):497–513. doi: 10.1007/s11126-025-10179-x (PMC12460516; doi:10.1007/s11126-025-10179-x)
Supplement: Supplementary file 1 — Supplementary Material 1 [file 11126_2025_10179_MOESM1_ESM.docx]

GANİME CAN GÜR

ASSOCIATE PROFESSOR

E-Mail Address	:	ganimegur@pau.edu.tr
Phone (Work)	:	2582964354-
Phone (Mobile)	:	5068556161
Address	:	PAMUKKALE ÜNİVERSİTESİ SAĞLIK BİLİMLERİ FAKÜLTESİ KINIKLI KAMPÜSÜ 20160 DENİZLİ

Education

Doctoral<br>2012<br>28/Ocak/2016	ATATURK UNIVERSITY->INSTITUTE OF HEALTH SCIENCES->PSYCHIATRIC NURSING (PhD)<br>Thesis name: Alkol kullanım bozukluğunda bilişsel davranışçı model temelli ve psikoeğitim destekli egzersizin depresyon, anksiyete ve öz yeterlilik düzeyine etkisi (2016) Thesis Advisor:(AYŞE OKANLI)
Master (Thesis)<br>2009<br>20/Şubat/2012	GAZIANTEP UNIVERSITY->INSTITUTE OF HEALTH SCIENCES->NURSING (MASTER) (WITH THESIS)<br>Thesis name: Madde bağımlılığı tanısı alan bireylerin sosyal işlevsellik ve içselleştirilmiş damgalanma düzeyleri (2012) Thesis Advisor:(DERYA TANRIVERDİ)
Bachelor's Degree<br>2005<br>25/Haziran/2009	GAZIANTEP UNIVERSITY->FACULTY OF HEALTH SCIENCES->DEPARTMENT OF NURSING->NURSING PR.

Academic Title

ASSOCIATE PROFESSOR<br>2021	PAMUKKALE UNIVERSITY/SAĞLIK BİLİMLERİ FAKÜLTESİ/HEMŞİRELİK BÖLÜMÜ/PSİKİYATRİ HEMŞİRELİĞİ ANABİLİM DALI
ASSISTANT PROFESSOR<br>2018	PAMUKKALE UNIVERSITY/SAĞLIK BİLİMLERİ FAKÜLTESİ/HEMŞİRELİK BÖLÜMÜ/PSİKİYATRİ HEMŞİRELİĞİ ANABİLİM DALI
RESEARCH ASSISTANT<br>2011-2018	ATATURK UNIVERSITY/SAĞLIK BİLİMLERİ FAKÜLTESİ/EBELİK BÖLÜMÜ

Supervised Theses:

Yüksek Lisans

2025

1. ÖZCAN İREM, (2025). Meme Kanseri Hastalarında Üst Bilişin, Anksiyete ve Depresyon Üzerindeki Etkisinde Şema Modlarının ve Belirsizliğe Toleransın Aracı Rolü, Pamukkale Üniversitesi->Sağlık Bilimleri Enstitüsü->Psikiyatri Hemşireliği Ana Bilim Dalı (Devam Ediyor)

2. DURUŞGAN ADALET, (2025). Çocukluk Çağı Travmaları, Nevrotik Kişilik Ozellikleri ve Olumsuz Bilişsel Duygu Düzenleme Arasındaki Etkileşimin Depresyon Üzerindeki Rolü: Ruminatif Düşünmenin Moderatörlüğünde Bir İnceleme, Pamukkale Üniversitesi->Sağlık Bilimleri Enstitüsü->Psikiyatri Hemşireliği Ana Bilim Dalı (Devam Ediyor)

3. OKUNAKOL BEHÇET KAGAN, (2025). Bilişsel Şemalar ile Somatoform Belirtiler Arasındaki İlişkide Savunma Mekanizmalarının Aracı Rolü, Pamukkale Üniversitesi->Sağlık Bilimleri Enstitüsü->Psikiyatri Hemşireliği Ana Bilim Dalı (Devam Ediyor)

2023

4. YURTSEVEN FATMA, (2023). Ruh Sağlığı Okuryazarlığı Ölçeği'nin Türkçe geçerlik ve güvenirlik çalışması, Pamukkale Üniversitesi->Sağlık Bilimleri Enstitüsü->Hemşirelik Ana Bilim Dalı (Tamamlandı)

Positions in Projects:

1. Hemşirelik Öğrencilerine Verilen Farkındalık Temelli Empati Eğitiminin Obez Hastalara Yönelik Tutum ve Empati Düzeyine Etkisi, TUBITAK Project (1002), Yürütücü:YILMAZ EMİNE,Araştırmacı:CAN GÜR GANİME, , 01/11/2020 - 01/11/2021 (NATIONAL)
2. Sedanter Bireyler İçin İnternet Tabanlı Egzersiz Eğitimi ve Reçetesi Programının Geliştirilmesi (ERVE Pro), TUBITAK Project (1002), Yürütücü:FATİH GÜR, Araştırmacı:GANİME CAN GÜR, Araştırmacı:VEDAT AYAN, , 15/10/2019 - 15/12/2020 (NATIONAL)

Administrative Position

Faculty Executive Board Membership<br>11.12.2022	PAMUKKALE UNIVERSITY->FACULTY OF HEALTH SCIENCES->DEPARTMENT OF NURSING/SAĞLIK BİLİMLERİ FAKÜLTESİ/HEMŞİRELİK BÖLÜMÜ
Institute Executive Board Membership<br>07.12.2022	PAMUKKALE UNIVERSITY->INSTITUTE OF HEALTH SCIENCES/SAĞLIK BİLİMLERİ ENSTİTÜSÜ
Commission Membership<br>2018	PAMUKKALE UNIVERSITY->FACULTY OF HEALTH SCIENCES->DEPARTMENT OF NURSING/SAĞLIK BİLİMLERİ FAKÜLTESİ/HEMŞİRELİK BÖLÜMÜ

Memberships to Scientific Organizations

1. Psikiyatri Hemşireleri Derneği, Member , 2012

Awards

1. The “Best of 2020 List” of Health Promotion Researchers, American Journal of Health Promotion, UNITED STATES OF AMERICA, 2021
2. TÜBİTAK tarafından verilen Uluslararası Bilimsel Yayınları Teşvik Ödülü (Is Exercise a Useful Intervention in the Treatment of Alcohol Use Disorder? Systematic Review and Meta-Analysis), 2020
3. TÜBİTAK tarafından verilen Uluslararası Bilimsel Yayınları Teşvik Ödülü (The Effects of Cognitive-Behavioral Model-Based Intervention on Depression, Anxiety, and Self-Efficacy in Alcohol Use Disorder), 2019
4. TÜBİTAK tarafından verilen Uluslararası Bilimsel Yayınları Teşvik Ödülü (Social Functioning and Internalized Stigma in Individuals Diagnosed with Substance Use Disorder), 2015

Works

Articles published in international refereed journals:

1. GÜR FATİH,CAN GÜR GANİME (2025). The relationship between physical activity and problematic internet use in Turkish college students: the chain-mediated role of self-control and distress. Psychiatric Quarterly (Publication No: 9529786)
2. CAN GÜR GANİME,ÖZGÜN ÖZTÜRK FATMA (2024). YÜRÜTÜCÜ İŞLEVLER ÖLÇEĞİNİN GELİŞTİRİLMESİ: GEÇERLİLİK GÜVENİRLİK ÇALIŞMASI. Karadeniz Uluslararası Bilimsel Dergi, 63, 31-50., Doi: 10.17498/kdeniz.1512772 (Publication No: 9148758)
3. YURTSEVEN FATMA,CAN GÜR GANİME (2024). Reliability and Validity of the Turkish Version of the Modified Mental Health Literacy Scale. CURRENT PSYCHOLOGY (Publication No: 9070178)
4. ÖZGÜN ÖZTÜRK FATMA,CAN GÜR GANİME (2024). Development and validation of the IS-C psychometric tool for evaluating children's impulsivity. International Journal of Assessment Tools in Education, 11(2), 388-405., Doi: 10.21449/ijate.1397644 (Publication No: 9339766)
5. CAN GÜR GANİME (2024). Shattered Ground, Resilient Souls: Examining Coping Strategies, Social Support, and Earthquake Exposure's Effects on Post-Traumatic Stress Disorder Symptoms Among Adolescent and Young Adults Survivors of the 2023 Earthquake in Türkiye. CURRENT PSYCHOLOGY, 43, 21745-21755., Doi: 10.1007/s12144-024-05995-6 (Publication No: 8964159)

Articles published in international refereed journals:

6. ÖZGÜN ÖZTÜRK FATMA, CAN GÜR GANİME (2024). Cross-cultural adaptation, reliability, and validity of the Turkish version of the smoking rationalization scale. Journal of Substance Use, 29(2), 209-215., Doi: 10.1080/14659891.2022.2144510 (Publication No: 7906813)
7. CAN GÜR GANİME, YILMAZ EMİNE (2024). Mindfulness-based empathy training supported by Obese Simulation Suit: Randomized Controlled Trial. Current Psychology, 43, 19532-19547., Doi: 10.1007/s12144-024-05719-w (Publication No: 8925403)
8. CAN GÜR GANİME, YILMAZ EMİNE (2023). Effectiveness of Interventions in Reducing Substance-Related Stigma: A Systematic Review and Meta-Analysis of Randomized Controlled Trials. ISSUES IN MENTAL HEALTH NURSING, 44(3), 14-175., Doi: 10.1080/01612840.2022.2163439 (Publication No: 8225872)
9. CAN GÜR GANİME, ÖZGÜN ÖZTÜRK FATMA (2023). Rumination, Basic Beliefs, and Posttraumatic Growth in Cancer Patients: A Moderated Mediation Model. Cancer Nursing, 48(3) (Publication No: 8460931)
10. CAN GÜR GANİME,TANRIVERDİ DERYA,Ariti Mahsun,ÖZGÜN ÖZTÜRK FATMA (2022). The Adaptation of the Substance Use Stigma Mechanism Scale (SU-SMS) Into Turkish: A Validity and Reliability Study. Journal of the American Psychiatric Nurses Association, 28(4), 295-305., Doi: 10.1177/1078390320949927 (Publication No: 6464498)
11. CAN GÜR GANİME, ALTINBAŞ YASEMİN (2022). Covid-19 Literacy Scale: Turkish Validity and Reliability Study. Clinical Nursing Research, 31(3), 404-412., Doi: 10.1177/10547738211059879 (Publication No: 7262425)
12. CAN GÜR GANİME, YILMAZ EMİNE (2021). The Effect of Intercultural Nursing Training on Nursing Students' Intercultural Sensitivity and Empathic Tendency Level: Randomised Controlled Trial. Gümüşhane Üniversitesi Sağlık Bilimleri Dergisi, 10(1), 130-137., Doi: 10.37989/gumussagbil.869660 (Publication No: 6997241)
13. CAN GÜR GANİME, YILMAZ EMİNE (2021). Tip 2 Diyabet Hastalarında Damgalama Değerlendirme Ölçeği'nin Türkçe Geçerlik ve Güvenirlik Çalışması. Türkiye Klinikleri Hemşirelik Bilimleri Dergisi, 13(3), 667-675., Doi: 10.5336/nurses.2020-78878 (Publication No: 6716042)
14. YILMAZ EMİNE, CAN GÜR GANİME (2021). Bir Kamu Hastanesine Başvuran Kadınların Doğum Eyleminde Doğum Korkusu ve Doğum Ağrısı Düzeyleri ile Etkileyen Faktörlerin Belirlenmesi. Türkiye Klinikleri Sağlık Bilimleri Dergisi, 6(4), 752-759., Doi: 10.5336/healthsci.2020-80882 (Publication No: 6948921)
15. CAN GÜR GANİME (2021). Psychometric Properties of the Turkish Version: The Challenges to Stopping Smoking (CSS-21) Scale. Journal of Substance Use, 26(1), 107-113., Doi: 10.1080/14659891.2020.1846090 (Publication No: 6573671)
16. GÜR FATİH, CAN GÜR GANİME, AYAN VEDAT (2020). The Effect of the ERVE Smartphone App on Physical Activity, Quality of Life, Self-Efficacy, and Exercise Motivation for Inactive People: A Randomized Controlled Trial. European Journal of Integrative Medicine, 39(101198), Doi: 10.1016/j.eujim.2020.101198 (Publication No: 6464506)
17. GÜR FATİH, CAN GÜR GANİME (2020). Is Exercise a Useful Intervention in the Treatment of Alcohol Use Disorder? Systematic Review and Meta-Analysis. American Journal of Health Promotion, 34(5), 520-537., Doi: 10.1177/0890117120913169 (Publication No: 6714988)
18. CAN GÜR GANİME, YILMAZ EMİNE (2020). The Effects of Mindfulness-Based Empathy Training on Empathy and Aged Discrimination in Nursing Students: A Randomised Controlled Trial. Complementary Therapies in Clinical Practice, 39(101140), Doi: 10.1016/j.ctcp.2020.101140 (Publication No: 6714870)
19. CAN GÜR GANİME,OKANLI AYŞE (2019). The Effects of Cognitive-Behavioral Model-Based Intervention on Depression, Anxiety, and Self-Efficacy in Alcohol Use Disorder. Clinical Nursing Research, 28(1), 52-78., Doi: 10.1177/1054773817722688 (Publication No: 7107523)
20. GÜR FATİH, CAN GANİME, OKANLI AYŞE (2017). The Effect of the Cognitive-behavioral Model-based Psychoeducation and Exercise Intervention on Quality of Life in Alcohol Use Disorder. Archives of Psychiatric Nursing, 31(6), 541-548., Doi: 10.1016/j.apnu.2017.07.005 (Publication No: 4068697)
21. CAN GANİME, YILMAZ EMİNE, ASİ KARAKAŞ SİBEL, POLAT HATİCE (2017). The Correlation Between Levels of Coping with Stress and Attitude Towards Smoking in Patients with Schizophrenia. International Journal of Caring Sciences, 10(1), 191-198. (Publication No: 7065803)
22. CAN GANİME, TANRIVERDİ DERYA (2015). Social Functioning and Internalized Stigma in Individuals Diagnosed with Substance Use Disorder. Archives of Psychiatric Nursing, 29(6), 441-446., Doi: 10.1016/j.apnu.2015.07.008 (Publication No: 2347988)
23. TANRIVERDİ DERYA, SAVAŞ ESEN, CAN GANİME (2012). Posttraumatic Growth and Social Support in Turkish Patients with Cancer. Asian Pacific Journal of Cancer Prevention, 13(9), 4311-4314., Doi: 10.7314/APJCP.2012.13.9.4311 (Publication No: 382010)

B. Papers presented at international scientific meetings and published in proceedings :

1. ÖZGÜN ÖZTÜRK FATMA, CAN GÜR GANİME (2022). Development Of A Psychometric Tool For Assessing Impulsivity In The Children: A Validity Reliability Study. KARADENIZ 11th INTERNATIONAL CONFERENCE ON APPLIED SCIENCES (Abstract/Oral Presentation) (Publication No: 7986830)
2. CAN GÜR GANİME, ALTINBAŞ YASEMİN (2021). Covid-19 Okuryazarlığı Ölçeği: Türkçe Geçerlik ve Güvenirlik Çalışması. 2. Uluslararası 3. Ulusal Halk Sağlığı Hemşireliği Kongresi/ 2nd International 3rd National Public Health Nursing Congress, 134 (Abstract/Oral Presentation) (Publication No: 6948962)
3. YILMAZ EMİNE, CAN GÜR GANİME (2020). Doğum Eyleminde Doğum Korkusu ve Doğum Ağrısının Belirlenmesi ve Etkileyen Faktörler. Gevher Nesibe 6. Uluslararası Sağlık Bilimleri Kongresi (Abstract/Oral Presentation) (Publication No: 6619356)
4. CAN GÜR GANİME (2020). Primer İnfertil Kadınların İnfertiliteye Bağlı Algıladıkları Damgalanma Düzeyleri ve Etkileyen Faktörlerin Belirlenmesi. 5th International Scientific Research Congress (IBAD-2020), 91 (Abstract/Oral Presentation) (Publication No: 6464596)
5. GÜR FATİH, CAN GANİME, OKANLI AYŞE (2015). The Effect of Cognitive Behavioral Model Based Exercise Practices on the Quality of Life in Alcohol Use Disorders. III. Internatıonal Exercıse and Sport Psychology Congress, 24 (Abstract/Oral Presentation) (Publication No: 1995755)
6. ŞAHİN ALTUN ÖZLEM, EJDER APAY SERAP, SÖNMEZ (SAKAR) TUĞÇE, Polat Ümran, Mehel Tutuk Pınar, Düzenli Zehra, Nazlı Ayşe, Aslan Rabia, CAN GANİME, YURTTAŞ AFİFE (2015). Assessment of the Mental State of Women in Turkey: A multi-center Cross-Sectional Study. The Care 4 International Scientific Nursing and Midwifery Congress (Abstract/Poster) (Publication No: 6949028)
7. TANRIVERDİ DERYA, CAN GANİME (2014). Posttravmatik Büyüme: Zorluklardan Güç Toplamak. III. Uluslararası VII. Ulusal Psikiyatri Hemşireliği Kongresi, 345 (Abstract/Poster) (Publication No: 7067786)
8. EKİNCİ MİNE, ŞAHİN ALTUN ÖZLEM, CAN GANİME (2013). Hemşirelik Öğrencilerinin Stresle Başa Çıkma Tarzları ve Atılganlık Düzeylerinin Bazı Değişkenler Açısından İncelenmesi. HORATIO, European Psychiatric Nursing Congress, 149 (Abstract/Poster) (Publication No: 383517)
9. YILDIZ ESRA, CENGİZ MİNE, CAN GANİME (2013). Baccalaureate and Masters' Degree Nursing Students' Levels of Critical Thinking. Horatio, European Psychiatric Nursing Congress, 199 (Abstract/Poster) (Publication No: 7067792)
10. CAN GANİME, TANRIVERDİ DERYA (2012). Madde Bağımlılığı Tanısı Alan Bireylerin Sosyal İşlevsellik ve İçselleştirilmiş Damgalanma Düzeyleri. II.Uluslararası VI. Ulusal Psikiyatri Hemşireliği Kongresi, 133-134. (Full Text Publication/Oral Presentation) (Publication No: 383078)

C. Authored national/international books or chapters in books:

C2. Chapters written in national/international books:

1. Turkiye Klinikleri Psychiatric Nursing-Special Topics, Chapter name:(Ruh Sağlığının Geliştirilmesi İçin Geleceğe Yönelik Öneriler) (2024)., CAN GÜR GANİME, Türkiye Klinikleri, Editor:TANRIVERDİ DERYA, Edition no:1, Pages 85, ISBN:978-625-395-399-7, Turkish (Scientific Book) (Publication No: 9244948)
2. KRONİK HASTALIKLARIN YÖNETİMİ VE KANITA DAYALI HEMŞİRELİK UYGULAMALARI, Chapter name:(Kronik hastalıkların psikososyal bakımı ve kanıta dayalı hemşirelik uygulamaları) (2022)., OKANLI AYŞE, CAN GÜR GANİME, Nobel Tıp Kitabevleri, Editor:Elanur YILMAZ KARABULUTLU, Gülcan BAHÇECİOĞLU TURAN, Edition no:1, Pages 447, ISBN:978-605-335-760-5, Turkish (Scientific Book) (Publication No: 7986806)
3. Farklı Yönleriyle Ruh Sağlığı ve Psikiyatri Hemşireliği, Chapter name:(Posttravmatik Büyüme: Zorluklardan Güç Toplamak) (2021)., CAN GÜR GANİME, TANRIVERDİ DERYA, Çukurova Nobel Tıp Kitabevi, Editor:Derya Tanrıverdi, Edition no:1, Pages 552, ISBN:978-605-2369-33-3, Turkish (Scientific Book) (Publication No: 6948952)
4. SAĞLIK & BİLİM: Hemşirelik, Chapter name:(Tedavinin Önündeki Engel: Damgalama ve Madde Kullanım Bozukluğu) (2021)., CAN GÜR GANİME, Efe Akademi Yayınları, Editor:GÜLCAN KENDİRKIRAN, Edition no:1, Pages 184, ISBN:978-625-7601-82-5, Turkish (Scientific Book) (Publication No: 7067566)
5. Farklı Yönleriyle Ruh Sağlığı ve Psikiyatri Hemşireliği, Chapter name:(Ruh Sağlığı Örgütlenmesi ve Politikaları) (2021)., CAN GÜR GANİME, TANRIVERDİ DERYA, Çukurova Nobel Tıp Kitabevi, Editor:DERYA TANRIVERDİ, Edition no:1, Pages 552, ISBN:978-605-2369-33-3, Turkish (Scientific Book) (Publication No: 7067547)
6. Pozitif Güçlenme ve İyi Hissetmek, Chapter name:(İyi Hissettirecek Sağlıklı Yaşam Egzersiz Reçeteleri) (2019)., GÜR FATİH, AYAN VEDAT, CAN GÜR GANİME, Türkiye Klinikleri, Editor:Ayşe Okanlı, Edition no:1, Pages 43, ISBN:978-605-7650-11-5, Turkish (Scientific Book) (Publication No: 5897392)

D. Articles published in national refereed journals :

1. CAN GANİME, EJDER APAY SERAP, ÖZORHAN ELİF YAĞMUR, OKANLI AYŞE, ŞAHİN ALTUN ÖZLEM (2015). Gebelik Hezeyanı. Literatür Sempozyum, 1(5), 21-30. (Control No: 1995243)
2. OKANLI AYŞE, CAN GANİME (2014). Bir Kuşaktan Diğerine Şiddet. Sağlıkla Hemşirelik Dergisi(12), 56-57. (Control No: 7066487)
3. EKİNCİ MİNE, ŞAHİN ALTUN ÖZLEM, CAN GANİME (2013). Hemşirelik Öğrencilerinin Stresle Başa Çıkma Tarzları ve Atılganlık Düzeylerinin Bazı Değişkenler Açısından İncelenmesi. Psikiyatri Hemşireliği Dergisi - Journal of Psychiatric Nursing, 4(2), 67-74., Doi: 10.5505/phd.2013.85856, Citation Counts: 3 (Control No: 383795)
4. TANRIVERDİ DERYA, SAVAŞ ESEN, GÖNÜLLÜOĞLU (UZDİL) NURCAN, KURDAL (BAŞKAYA) EBRU, CAN GÜR GANİME (2011). Lise Öğrencilerinin Yeme Tutumları, Yeme Davranışları ve Benlik Saygılarının İncelenmesi. Gaziantep Tıp Dergisi, 17(1), 33-39. (Control No: 7067858)

E. Papers presented at national scientific meetings and published in

1. CAN GANİME, YILMAZ EMİNE, ASİ KARAKAŞ SİBEL (2015). Şizofreni Hastalarında Sigaraya Karşı Tutum ve Stresle Başa Çıkma Düzeyleri Arasındaki İlişkinin Belirlenmesi . 15. Ulusal Hemşirelik Kongresi (Abstract/Poster) (Publication No: 1995855)
2. CAN GANİME, EJDER APAY SERAP, ÖZORHAN ELİF YAĞMUR, OKANLI AYŞE, ŞAHİN ALTUN ÖZLEM (2013). Gebelik Hezeyanı. 14. Ulusal Hemşirelik Kongresi (Uluslararası Katılımlı), 217 (Abstract/Poster) (Publication No: 382852)

Non-academic Experience

2010-2011	**Hemşire**	Adıyaman Üniversitesi Eğitim ve Araştırma Hastanesi, Psikiyatri Servisi, Hemşire, (Public)
2009-2010	**Hemşire**	Gaziantep Üniversitesi Onkoloji Hastanesi, Yetişkin Onkoloji Servisi, Hemşire , (Public)

Certificate

564115	Cinsel Danışmanlık Eğitimi, Cinsel Danışmanlık Eğitimi, Psikoaktif, Certificate, 30.10.2022 -31.01.2023 (National)
564117	Evlilik Hukuku ve Aile Arabuluculuğu Eğitimi, Evlilik Hukuku ve Aile Arabuluculuğu Eğitimi, Psikoaktif, Certificate, 30.10.2022 -31.01.2023 (National)
564113	Aile Danışmanlığı, Aile Danışmanlığı, ESENYURT ÜNİVERSİTESİ SÜREKLİ EĞİTİM MERKEZİ, Certificate, 30.10.2022 -31.01.2023 (International)
564116	Çift Danışmanlığı Eğitimi, Çift Danışmanlığı Eğitimi, Psikoaktif, Certificate, 30.10.2022 -31.01.2023 (National)
401975	Uzaktan Eğitim Yetkinlik Sertifikası, YÖKAK ve SABAK, ONLİNE, Certificate, 20.10.2020 -01.11.2020 (National)
193651	“2. Ulusal İntegratif Tıp Kongresi", İntegratif Tıp Derneği, ANTALYA, Certificate, 13.11.2015 -15.11.2015 (National)
193647	“VI. International Art Therapy Congress”, Atatürk Üniversitesi Kazım Karabekir Eğitim Fakültesi, ERZURUM, Certificate, 05.06.2015 -06.06.2015 (International)
193646	“13. Uluslararası Spor Bilimleri Kongresi”, Selçuk Üniversitesi ve Spor Bilimleri Derneği, İSTANBUL, Certificate, 07.11.2014 -09.11.2014 (International)
193664	“16. Üniversitelerarası İşbirliği Programı (ÜNİP) Toplantısı”, Üniversitelerarası İşbirliği Programı (ÜNİP) ve Atatürk Üniversitesi, ERZURUM, Certificate, 09.05.2014 -09.05.2014 (National)

Course

401974	Güç Analizi, Pamukkale Üniversitesi , DENİZLİ, Course, 02.04.2021 -02.04.2021 (National)
193658	“Akademik Düzeyde Tübitak Proje Hazırlama Eğitimi”, Atatürk üniversitesi Proje geliştirme ve Koordinasyon Ofisi ve TÜBİTAK, ERZURUM, Course, 15.04.2015 -17.05.2015 (National)

193662 “İş Sağlığı ve Güvenliği Eğitimi”, Atatürk Üniversitesi Sağlık Bilimleri Fakültesi, ERZURUM, Course, 22.04.2014 -27.04.2014 (National)

193659 “Hipnoz Eğitimi Kursu”, Aile Hekimliği Okulu Derneği, ERZURUM, Course, 21.03.2014 -18.04.2014 (National)

193657 “Dikkat Eksikliği ve Hiperaktivite Bozukluğu Kursu”, Türkiye Psikiyatri Derneği ve Atatürk Üniversitesi Tıp Fakültesi Psikiyatri Anabilim Dalı, ERZURUM, Course, 16.03.2013 -16.03.2013 (National)

193654 “Temel Araştırma Planlama, Çözümleme ve Geçerlilik Güvenirlik Analizleri Kursu”, III. International VII. National Psychiatric Nursing Congress
Atatürk Üniversitesi Sağlık Bilimleri Fakültesi ve Psikiyatri Hemşireleri Derneği, ERZURUM, Course, 04.10.2011 -07.10.2011 (National)

193653 ‘Yara Bakımında Güncel Yaklaşımlar Kursu”, Harran Üniversitesi ve Şanlıurfa Valiliği İl Sağlık Müdürlüğü, ŞANLIURFA, Course, 06.05.2011 -06.05.2011 (National)

193652 “Beyin Yapısı ve İşlevi Kursu, Gaziantep Üniversitesi Tıp Fakültesi Psikiyatri Anabilim Dalı ve Psikofarmakoloji Derneği, GAZİANTEP, Course, 26.04.2010 -02.05.2010 (National)

Workshop

193668 ‘‘8. Engelsiz Üniversiteler Çalıştayı (Uluslararası Katılımlı)”, Atatürk Üniversitesi Rektörlüğü ve Engelsiz Üniversite Birimi, ERZURUM, Workshop, 15.05.2014 -17.05.2014 (National)

193666 ‘‘Ruh Sağlığı ve Psikiyatri Hemşireliği Eğitimi ve Uygulaması Çalıştayı- III”, Psikiyatri Hemşireleri Derneği, İSTANBUL, Workshop, 30.10.2013 -30.10.2013 (National)

Seminar

405231 Davranışsal Bağımlılık Eğitimi, 2018-2023 Uyuşturucu ile Mücadele Ulusal Strateji Belgesi ve Eylem Planı kapsamında 1.,2., 3. ve 4. sınıf hemşirelik öğrencilerine tarafımdan verilen eğitim, Pamukkale Üniversitesi, Sağlık Bilimleri Fakültesi, Seminar, 08.03.2021 -10.03.2021 (National)

Product

404956 ERVE, MOBİL APP (Araştırmacıların kendi imkanlarıyla finanse ettiği), ANDROID PLAY ve APPLE STORE
https://play.google.com/store/apps/details?id=com.erve&hl=en_US&gl=US, Product, 24.02.2019 (National)

401981 ERVE Pro, TUBITAK , APP STORE, ANDROID STORE
https://ervepro.com/about
https://apps.apple.com/tr/app/erve-pro/id1517349478
https://apk-andriod.com/androidapk/app/1517349478/erve-pro, Product, 19.02.2021 (International)
